# New Insights into Antioxidant Peptides: An Overview of Efficient Screening, Evaluation Models, Molecular Mechanisms, and Applications

**DOI:** 10.3390/antiox13020203

**Published:** 2024-02-05

**Authors:** Yuhao Zhang, Yun Li, Zhengze Quan, Ping Xiao, Jin-Ao Duan

**Affiliations:** Jiangsu Collaborative Innovation Center of Chinese Medicinal Resources Industrialization, National and Local Collaborative Engineering Center of Chinese Medicinal Resources Industrialization and Formulae Innovative Medicine, and Jiangsu Key Laboratory for High Technology Research of TCM Formulae, Nanjing University of Chinese Medicine, Nanjing 210023, China; zhangyuhao@njucm.edu.cn (Y.Z.); liyun@njucm.edu.cn (Y.L.); quanzhengze@njucm.edu.cn (Z.Q.)

**Keywords:** antioxidant peptides, novel technologies, efficient screening, metal chelating peptides, antioxidant signaling pathways, application, collagen peptides

## Abstract

Antioxidant peptides are currently a hotspot in food science, pharmaceuticals, and cosmetics. In different fields, the screening, activity evaluation, mechanisms, and applications of antioxidant peptides are the pivotal areas of research. Among these topics, the efficient screening of antioxidant peptides stands at the forefront of cutting-edge research. To this end, efficient screening with novel technologies has significantly accelerated the research process, gradually replacing the traditional approach. After the novel antioxidant peptides are screened and identified, a time-consuming activity evaluation is another indispensable procedure, especially in in vivo models. Cellular and rodent models have been widely used for activity evaluation, whilst non-rodent models provide an efficient solution, even with the potential for high-throughput screening. Meanwhile, further research of molecular mechanisms can elucidate the essence underlying the activity, which is related to several signaling pathways, including Keap1-Nrf2/ARE, mitochondria-dependent apoptosis, TGF-β/SMAD, AMPK/SIRT1/PGC-1α, PI3K/Akt/mTOR, and NF-κB. Last but not least, antioxidant peptides have broad applications in food manufacture, therapy, and the cosmetics industry, which requires a systematic review. This review introduces novel technologies for the efficient screening of antioxidant peptides, categorized with a new vision. A wide range of activity evaluation assays, encompassing cellular models, as well as rodent and non-rodent models, are provided in a comprehensive manner. In addition, recent advances in molecular mechanisms are analyzed with specific cases. Finally, the applications of antioxidant peptides in food production, therapy, and cosmetics are systematically reviewed.

## 1. Introduction

The human body maintains a balance between oxidation and antioxidant defense under normal conditions. However, the balance can be interrupted when there is excessive production of reactive oxygen species (ROS) due to exogenous factors (UV light, ionizing radiation, industrial toxins, tobacco smoke) or endogenous ones (inflammation and immune response, metabolism), thus causing oxidative stress (OS) [1]. Most ROS are generated in mitochondria during the course of the electron transport chain (ETC) [2]. This includes a range of oxygen radical species (O_2_^•−^, HO^•^, RO^•^, and ROO^•^) and non-radical species (^1^O_2_, H_2_O_2_, and HOCl) [3]. The excessive amount of ROS can lead to DNA base destruction, protein carbonylation, and lipid peroxidation, which in turn leads to a series of diseases, such as cardiovascular diseases, neurodegenerative diseases, and atherosclerosis [4]. Fortunately, antioxidants, in particular antioxidant peptides, provide a good solution to alleviate the impacts of OS. Based on the specific amino acid sequence, spatial structure, and small molecule (<6 kDa) properties, antioxidant peptides can scavenge ROS efficiently [5]. In addition, food-derived antioxidant peptides are available from a variety of sources, including plant, animal, and dairy proteins, which represents a sustainable, environmentally friendly, and safe extraction strategy [6]. To discover novel antioxidant peptides, the traditional approach involves obtaining hydrolysates from food-derived proteins by enzymatic hydrolysis or fermentation. Commercial enzymes can directly hydrolyze proteins to produce antioxidant peptides. During fermentation, microorganisms can secrete proteases that hydrolyze proteins to produce antioxidant peptides [7]. Screening strains with specificity and higher proteolytic activity is another step worth considering [8]. Complex isolation, purification, and identification steps (or direct identification, but lack of information on the composition of the short peptides) are still in need [8,9]. The laborious protocol has become a hindrance on the route to efficient screening. To this end, our previous publication introduced eight novel and efficient techniques for the discovery of antioxidant peptides [10]. Nevertheless, there are currently more cases of novel technologies in the efficient screening of antioxidant peptides that deserve to be reported. In this review, a new categorization vision is provided, including free radical scavenging, metal ion chelation, and the essential target of antioxidant peptides, Kelch-like ECH-associated protein 1/NFE2-related factor 2 (Keap1-Nrf2) interaction. From these three perspectives, the current applications of novel technologies in efficient screening are critically reviewed.

Activity evaluation is an indispensable step for the identified peptides. Conventional in vitro chemical evaluations, including free radical scavenging, metal ion chelating, and lipid peroxidation inhibition (LPI) capacity assays, have been extensively reported and reviewed [11]. However, it is difficult for these assays to simulate the real physiological environment of the human body, and to some extent, the single chemical evaluation is unconvincing. In vitro cellular and in vivo rodent models have been widely used in the evaluation of antioxidant peptides, whilst the maintenance cost, as well as ethical issues, remain a concern for researchers. Non-rodent models, including zebrafish, *C. elegans*, and *Drosophila*, have been frequently used for evaluation in recent years. These models share the advantages of small size, low maintenance costs, and few ethical concerns [12]. Notably, the embryos and larvae of zebrafish and *C. elegans* are transparent throughout the body, thus enabling direct in vivo ROS detection with various fluorescent probes [13,14]. Meanwhile, human aging is closely associated with OS [15]. Therefore, *Drosophila* and *C. elegans* can be a great choice for evaluating the anti-aging effects of antioxidant peptides due to their short life cycle and lifespan [16,17]. More importantly, these non-rodent models have the potential to be developed into high-throughput drug screening platforms [18], which may enable the efficient screening of antioxidant peptides in the future.

In addition, mechanism research is usually carried out hand in hand with activity evaluation to reveal the nature of antioxidant peptide activity. Various molecular signaling pathways elucidate the different mechanisms of action underlying antioxidant peptides. Kelch-like ECH-associated protein 1/NFE2-related factor 2/antioxidant response elements (Keap1-Nrf2/ARE), mitochondria-dependent apoptosis, transforming growth factor-β/small mothers against decapentaplegic (TGF-β/SMAD), AMP-activated protein kinase/Sirtuin/peroxisome proliferator-activated receptor γ coactivator-1 α (AMPK/SIRT1/PGC-1α), phosphatidylinositol 3-kinase/Akt/the mammalian target of rapamycin (PI3K/Akt/mTOR), and nuclear factor-κB (NF-κB) are the six signaling pathways that can demonstrate the different molecular mechanisms of antioxidant peptides [19,20,21,22,23,24,25].

Finally, the application of antioxidant peptides focuses on food manufacturing, therapy, and the cosmetic industry. A systematic review is urgently required to summarize the applications of antioxidant peptides. This review focuses on the specific products of antioxidant peptides in food manufacture. Moreover, food-derived and synthetically designed antioxidant peptides possess different characteristics in therapy, so a comparison is needed. Finally, the applications of antioxidant peptides in the cosmetic industry focus on L-carnitine, glutathione (GSH), and collagen peptides, which have been widely used.

This review summarizes the advances of antioxidant peptides from four consecutive perspectives: screening-activity-mechanism-application. It provides current gaps in the research of antioxidant peptides through reasonable comparison and analysis. The purpose of this review is to provide new insights into the research of antioxidant peptides, as well as to provide directions for future research.

## 2. Novel Technologies in the Efficient Screening of Antioxidant Peptides

### 2.1. Efficient Screening Based on Free Radicals Scavenging

Screening antioxidant peptides based on free radical scavenging is currently assisted with various in silico tools, which can predict the quantitative structure-activity relationship (QSAR) of the peptides. Take the 3D-QSAR as an example. 3D-QSAR is an extension of traditional QSAR analysis (Hansch analysis and Free-Wilson analysis), which takes the critical property, the 3D structure of the molecule, as an essential element for activity prediction [26]. Peptides with outstanding free radical scavenging activity can be efficiently screened with 3D-QSAR technology. For instance, 107 tripeptides that were reported to scavenge ABTS^•+^ were initially collected from the online database as the candidates. After structure construction and energy minimization, peptide alignment, modeling (CoMSIA method), and verification, the contribution of peptide properties to the antioxidant activity was analyzed in the following order: spatial (41.4%) > aryl ring factor (15.3%) > hydrogen bond donor factor (14.2%) > hydrophobic (12.0%) > hydrogen bond acceptor factor (11.4%) > electrostatic (5.7%). Based on the result, two core tripeptide fragments, Pro-Trp-Leu (OH) and Pro-Trp-Leu (NH_2_), were obtained [27].

In addition to 3D-QSAR, other novel QSAR approaches are emerging, representing more comprehensive and in-depth research. The vector of radial distribution function descriptors and geometrical descriptors (SVRG)-DPPH^•^ and the vector of principal component score for electronic eigenvalue descriptors (SVEEVA)-DPPH^•^ models were constructed using stepwise regression to select the variables and the partial least squares model. Finally, three novel antioxidant peptides AGWACLVG (Ala-Gly-Trp-Ala-Cys-Leu-Val-Gly), YPLDL (Tyr-Pro-Leu-Asp-Leu), and IDLAY (Ile-Asp-Leu-Ala-Tyr) from *Auxenochlorella pyrenoidosa* were successfully characterized [28]. In contrast, the abovementioned 3D-QSAR case was performed on the prerequisite that the peptide sequence length was fixed (tripeptide). However, the peptide fractions extracted from food-derived protein matrices usually have different sequence lengths. In the case of *Auxenochlorella pyrenoidosa*, a two-terminal position numbering method was used, which can translate peptides of different lengths to the same [29]. This method provides insights into the use of QSAR for predicting food-derived antioxidant peptides based on free radical systems.

Finally, regarding in vitro techniques, real-time online detection provides new insights into the efficient screening of free radical scavenging peptides. Specifically, antioxidants can be detected and screened using high-performance liquid chromatography (HPLC) separation combined with free radical-packed columns. With HPLC-radical coupling, the antioxidant candidates undergo a post-column reaction with the corresponding free radicals, which decreases the absorbance at a specific wavelength. Each antioxidant separated by the HPLC column can be tested for its related antioxidant activity by observing the negative peaks on its profile. Finally, quantitative analysis can be achieved by the linear dependence of the negative peak area on the concentration of the reference antioxidant [30]. The technique has been applied to detect various food-derived antioxidants [31,32,33]. A novel free radical scavenging peptide FSDIPNPIGSENSEKTTMPLW (Phe-Ser-Asp-Ile-Pro-Asn-Pro-Ile-Gly-Ser-Glu-Asn-Ser-Glu-Lys-Thr-Thr-Met-Pro-Leu-Trp) has been isolated from bovine skim milk through HPLC-ABTS^•+^ detection [34].

### 2.2. Efficient Screening Based on Metal Ions Chelation

Chelating metal ions is a crucial activity of antioxidant peptides, of which the peptides with prominent activity in this regard are called metal chelating peptides (MCPs). Some state-of-the-art technologies have shown efficient performance in screening MCPs. To this end, surface plasmon resonance (SPR) and SwitchSENSE ^®^ technologies have been reviewed in our previous publication [10]. Both are characterized by high sensitivity and rapidity. Interestingly, efficient screening of MCPs is not limited to the two technologies instead.

A metal-organic framework (MOF) has the edge of a large specific surface area, a regular porous structure, and customizable porosity [35]. Among them, MIL-53 is constructed by chains of angle-sharing metal clusters connected by terephthalate ligands, which are massively chemically stable [36]. Significantly, it can efficiently and selectively enrich MCPs from complex protein hydrolysates. Based on the MOF, the researchers developed an attractive “angling” assay to screen antioxidant peptides. Specifically, the researchers synthesized and collected MIL-53(Cr), known as “bait”. The prepared rice dreg protein hydrolysate (RDPH) is the “fish pond”, where the MCPs are the “fish of interest”. The MIL-53(Cr) was mixed with RDPH, enriched, and centrifuged while waiting for the “angling”. Finally, the deposited MIL-53(Cr)-peptides are collected and eluted to obtain the desired Cr^3+^-chelating peptides. Two novel antioxidant peptides, GDMNP (Gly-Asp-Met-Asn-Pro) and LLLRW (Leu-Leu-Leu-Arg-Trp) were identified in this way [37]. The critical step is to design the “lure” in the “angling” process. Even though it has been proven valid in the case of MIL-53(Cr) in RDPH, more research is needed to verify the feasibility so the extrapolation of the protocol can be further facilitated.

Phage display was discussed in the previous publication to screen peptides based on the protein-protein interaction (PPI) inhibition of Keap1-Nrf2. The peptides of interest were progressively eluted by three to four rounds of biopanning with varying stringency [38]. Interestingly, the researcher has developed a new approach to screen MCPs with phage display. To be specific, Cu^2+^ immobilized resin was set as the immobilized ligand. The reverse biopanning was first performed to remove the phage bound on the resin, and then the Cu^2+^ affinity biopanning was carried out with TBST buffer, EDTA solution, and citrate buffer solution, respectively. The Cu^2+^ chelating peptides with the strongest affinity were gradually screened. Finally, a new antioxidant peptide, SAQIAPH (Ser-Ala-Gln-Ile-Ala-Pro-His), was identified. It can inhibit Cu^2+^-induced Aβ142 aggregation and attenuated Cu^2+^-mediated OS in N2a-sw cells, demonstrating the potential for the treatment of AD [39].

In addition, an integrated protocol was constructed to screen Fe^2+^ chelating peptides from complex hydrolysates with ion-pair reversed-phase liquid chromatography (IP-RPLC) and high-resolution mass spectrometry (HRMS). Simple, rapid, and sensitive screening can be achieved based on IP-RPLC-HRMS online data analysis of filtration in Fe^2+^-added peptide mixtures versus unadded controls [40]. This approach completes the transcendence to traditional immobilized metal ion affinity chromatography (IMAC) screening and builds a complete, applicable, efficient model.

Metal ions generally play a catalytic role in the OS reaction chain, and screening MCPs is essentially seen as an indirect strategy. Several rapid in vitro assays are still required in the process of screening. In addition, the analysis of critical amino acids in MCPs, such as His and Trp, cannot be overlooked, as both are characterized by a strong ability to form complexes with various metal ions [41].

### 2.3. Efficient Screening Based on the Protein-Protein Interaction (PPI) Inhibition of Keap1-Nrf2

The Keap1-Nrf2 is an essential and classical de-inhibitory antioxidant pathway [42]. Currently, most known Nrf2 activators are indirect inhibitors of the Keap1-Nrf2 PPI, functioning by modifying the sulfhydryl group on the cysteine of Keap1. However, this can lead to an “off-target” phenomenon without specific selection [43]. In contrast, it is an efficient approach to screen antioxidant peptides through the PPI inhibition of Keap1-Nrf2, which directly targets the Kelch domain of Keap1.

The following research is illustrated as an application in food science. Firstly, the antioxidant peptides were collected from an egg-derived proteins database, of which the binding ability to the Keap1 ligand was investigated by molecular docking. Twenty candidate peptides were left over in this procedure. Next, competitive analysis was performed with fluorescence polarization (FP), one of the most common techniques in screening PPI inhibitors of Keap1-Nrf2. It enables a rapid, sensitive analysis of intermolecular interactions in a non-cellular environment [44]. With the FITC-labelled 9-mer Nrf2 peptides containing the ETGE motif, 20 candidates were analyzed for competitive inhibition of Nrf2 binding to the Kelch domain of Keapl. Two antioxidant peptides, DKK (Asp-Lys-Lys) and DDW (Asp-Asp-Trp) were picked out by comparing the dissociation constant K_d_ with the binding curves. They both significantly reduced the structure of the FITC-labelled Nrf2 peptide with the Keap1 Kelch domain, suggesting their potent ability to inhibit direct PPI [45].

PPI modulators are small molecules that can interact with the protein-protein interface (or allosteric sites). It can inhibit the protein-protein interaction (functioning as PPI inhibitors), but it can stabilize or even enhance it as well [46]. Developing targeted peptides based on PPI interfacial interactions is the primary approach in developing PPI modulators. Interestingly, the Keap1-Nrf2 interfacial contact area is closer to small molecule-protein interactions (~300–1000 Å^2^), smaller than the common PPI-contacting interfacial area (~1500–3000 Å^2^). It provides a basis for rationalizing the synthesis of small-molecule peptide drugs [47]. Currently, the development of inhibitors of PPI based on Keap1-Nrf2 is mainly focused on synthetic peptides, with research areas favoring the pharmaceutical field. Research in this area progresses rapidly, and many efficient techniques are being developed. As in the previous case, FP was a tool in the early stages of developing Keap1-Nrf2 PPI inhibitors [48]. Apart from this, a fluorescence resonance energy transfer (FRET) assay is developed based on a neighboring fluorescence pathway, and a more optimized time-resolved fluorescence resonance energy transfer (TR-FRET) has been further developed [49]. SPR techniques developed based on competitive binding are also commonly used in this direction [50]. These techniques are always under constant optimization and updating. An ELISA-based technique has recently been developed for screening Keap1-Nrf2 PPI inhibitors [51]. In addition, other researchers have developed cellular screening strategies based on FRET and bimolecular fluorescence complementation (BiFC) techniques, which enabled the development of Keap1-Nrf2 PPI inhibitors in an intact cellular environment [52]. Finally, numerous in silico screenings are much more up-to-date and represent a more efficient and cost-effective strategy [53].

In this rapidly iterative research environment, a plethora of novel Keap1-Nrf2 PPI inhibitory peptides (antioxidant peptides) have been identified. The cyclic antioxidant peptides were developed based on a head-tail linkage strategy, which has effectively improved stability and membrane permeability and has been shown to have a 15-fold increase in potency compared to the counterpart linear peptide [54]. These antioxidant peptides developed based on artificial design, synthesis, and modification differ from how food-derived proteins are extracted and screened. To be specific, most synthetic peptides can be designed based on the minimal Nrf2 active sequences, ETGE motif, and DLG motif, and antioxidant peptides can be developed by directly utilizing a head-to-tail linked cyclic strategy, as well as coupling cell-penetrating peptides [47]. In other words, it is an ab initio design approach. In contrast, food-derived antioxidant peptides have to be screened from complex hydrolysates, where the PPI inhibition strategy mainly focuses on post-screening. In the case of egg-derived peptides, synthetic peptides are prepared for screening owing to an available virtual library of egg-derived proteins. Such an approach is distinct from screening unknown peptides from natural products, which have not been subjected to sequence identification.

It is recognized that food-derived antioxidant peptides are more environmentally friendly and less costly than artificially synthesized and designed peptides. Food-derived antioxidant peptides feature a sustainable mode of exploration and relatively lower toxicity [6]. Claiming antioxidant peptides from food proteins is a well-established approach. For Keap1-Nrf2, a critical antioxidant pathway, most current research on food-derived antioxidant peptides focuses on later elucidating their mechanism of action (or activity verification). The Keap1-Nrf2 PPI screening of food-derived antioxidant peptides requires more research efforts in this direction to “reverse the use” of this critical antioxidant pathway.

## 3. Evaluation Models

### 3.1. In Vitro Cellular Antioxidant Evaluation Models

In vitro cellular models are widely used in the activity evaluation of antioxidant peptides. The regular protocol is to construct OS cellular models and investigate the cytoprotective effects of antioxidant peptides. Rat hepatocytes and pheochromocytoma cells (PC12), mouse macrophages (RAW264.7), human epithelial cell line (Caco-2), and human umbilical vein endothelial cells (HUVECs) are the common cellular models for evaluation. Each can be applied in different situations, as the following sections illustrate.

#### 3.1.1. Rat Hepatocytes and Pheochromocytoma Cells (PC12)

The PC12 cell line is commonly used to construct OS models to study the neuroprotective effects of antioxidant peptides. For example, an antioxidant peptide brevinin-1FL, FWERCSRWLLN (Phe-Trp-Glu-Arg-Cys-Ser-Arg-Trp-Leu-Leu-Asn), was identified from the skin of *Fejervarya limnocharis*. It could exert neuroprotective effects in PC12 cells by reducing ROS levels, enhancing the activity of antioxidant enzymes, and inhibiting H_2_O_2_-induced apoptosis [55]. Similarly, three peptide fractions, QGRPWG (Gln-Gly-Arg-Pro-Trp-Gly), PSRADIY (Pro-Ser-Arg-Ala-Asp-Ile-Tyr), and AYNIPVNIAR (Ala-Tyr-Asn-Ile-Pro-Val-Asn-Ile-Ala-Arg) were identified from walnut protein hydrolysate. Among them, AYNIPVNIAR showed the strongest neuroprotective effects against H_2_O_2_-induced OS in PC12 cells in a dose-dependent manner [56].

#### 3.1.2. Mouse Macrophages (RAW264.7)

The murine stable cell line RAW264.7 can be used to evaluate the activity of antioxidant peptides. For instance, collagen peptides were prepared from the hydrolysis of sea cucumber protein. The collagen peptides could significantly promote the proliferation of RAW264.7 and reduce the H_2_O_2_-induced OS levels in RAW264.7. Meanwhile, the levels of SOD and GPx were increased, with the MDA levels decreased in RAW264.7 owing to the treatment of collagen peptides. These results demonstrated the protective effects of the sea cucumber-derived collagen peptides from OS in RAW264.7 cells [57]. Similarly, two antioxidant peptides, SDLKHFPF (Ser-Asp-Leu-Lys-His-Phe-Pro-Phe) and SDIKHFPF (Ser-Asp-Ile-Lys-His-Phe-Pro-Phe), were identified from *T. matsutake*. They could exert protective effects by ameliorating ROS levels and increasing the Bcl-2/Bax ratio (Bcl-2 is anti-apoptosis while Bax is pro-apoptosis) in RAW264.7 [58].

#### 3.1.3. Human Epithelial Cell Line (Caco-2)

The human epithelial cell line (Caco-2) is often used to evaluate the activity of antioxidant peptides. For instance, antioxidant peptides were obtained from the simulated gastrointestinal digestion (INFOGEST method) of whey proteins. The peptide digests decreased the ROS levels in H_2_O_2_-exposed Caco-2 intestinal cells, demonstrating the cytoprotective effects of whey peptides in the gastrointestinal system [59]. Moreover, research on the epithelial transport of antioxidant peptides can be conducted in Caco-2 cells. For example, two antioxidant peptides, YFCLT (Tyr-Phe-Cys-Leu-Thr) and GLLLPH (Gly-Leu-Leu-Leu-Pro-His), were identified from corn gluten. Both peptides could transport across the Caco-2 cell monolayers in complete form [60]. The transepithelial peptides can be further screened through in vitro antioxidant activity assays [61]. This way, antioxidant peptides with prominent absorption and bioavailability can be obtained.

#### 3.1.4. Human Umbilical Vein Endothelial Cells (HUVECs)

HUVECs, isolated from the human umbilical cord, are one of the major constituent cells of the umbilical vein and are commonly used for research on vascular endothelial cell function. An antioxidant peptide, WDHHAPQLR (Trp-Asp-His-His-Ala-Pro-Gln-Leu-Arg), was identified from rapeseed protein. It exerted protective effects in H_2_O_2_-exposed HUVECs by inhibiting apoptosis. Specifically, WDHHAPQLR could down-regulate the expression of Bax and caspase-3 while up-regulating the expression of Bcl-2 in H_2_O_2_-treated HUVECs [62]. Similarly, three antioxidant peptides GEYGFE (Gly-Glu-Tyr-Gly-Phe-Glu), PSVSLT (Pro-Ser-Val-Ser-Leu-Thr), and IELFPGLP (Ile-Glu-Leu-Phe-Pro-Gly-Leu-Pro) were characterized from Siberian sturgeon (*Acipenserbaerii*) cartilages. GEYGFE and IELFPGLP showed cytoprotective effects in the H_2_O_2_-exposed HUVECs by enhancing the activity of SOD and GPx and decreasing the levels of ROS and MDA [63].

### 3.2. In Vivo Activity Evaluation in Rodent and Non-Rodent Models

#### 3.2.1. Rodent Models

Mice and rats are the commonly used rodent models to evaluate the activity of antioxidant peptides. AS OS is closely associated with human diseases, the potential therapeutic effects of antioxidant peptides can be tested by constructing relevant models in mice or rats.

The obese mice model can be used to evaluate the anti-obesity and anti-hyperglycemic effects of antioxidant peptides. For instance, *Meretrix lusoria* protamex hydrolysate (MPLH) treatment increased hepatic GSH levels and antioxidant enzymes (GPx, glutathione reductase, SOD, and CAT) activity in a dose-dependent manner compared to the control group. The impaired glucose tolerance in ob/ob control mice was repaired. Meanwhile, MLPH significantly inhibited adipogenesis and hyperglycemia-related mRNA expression. These results demonstrate the anti-obesity and anti-hyperglycemia potential of MLPH [64]. Similarly, the streptozocin-induced type 1 diabetes mice were used to evaluate the activity of *Harpadon nehereus* bones collagen peptide (HNCP). HNCP treatment increased the levels of CAT, SOD, and GPx in diabetes mice. Meanwhile, blood glucose, triglyceride (TG), and low-density lipoprotein cholesterol levels decreased while serum insulin and hepatic glycogen levels increased. These results illustrate the potential of HNCP to improve glucose-lipid metabolism and lower blood glucose [65].

Cases are similar in rat models. To evaluate the activity of an antioxidant peptide, VNP (Val-Asn-Pro), an OS model was constructed in AAPH-exposed rats. The results showed that VNP could increase the activity and gene expression of CAT, GPX1, SOD1, and HO-1 through the Nrf2/Keap1-p38MAPK/PI3K-MafK signaling pathway [66]. Moreover, spontaneously hypertensive rats (SHRs) were used to determine the activity of different antioxidant peptide fractions from wheat bran protein hydrolysate (WPH). The results showed that the peptide fraction (<1 kDa) had a higher ACE and renin inhibition rate and oxygen radical antioxidant activity than WPH and other fractions. Meanwhile, a similar edge could remain in the blood pressure-lowering effects. To sum up, WPH, as well as its ultrafiltration fractions (especially < 1 kDa), has excellent potential to be the source of antihypertensive and antioxidant peptides [67].

#### 3.2.2. Non-Rodent Models

Compared to rodent models, the non-rodent models normally have fewer ethical concerns. Three non-rodent models, *Danio rerio*, *Drosophila melanogaster*, and *Caenorhabditis elegans*, have the advantage of being small, easy to culture, and simplified for experimental manipulation [68]. Constructing OS models based on these three models, combined with the detection of OS/antioxidant biomarkers, provides a solution for evaluating the activity of antioxidant peptides. More importantly, the non-rodent models have the potential to be developed and integrated into a high-throughput screening platform, which represents the future research direction for antioxidant peptides.

##### Danio Rerio (Zebrafish)

Zebrafish is a commonly used vertebrate non-rodent model organism. Embryonic or larval zebrafish are naturally transparent. Therefore, three fluorescent probe dyes, acridine orange (AO), 2′,7′-dichlorodihydrofluorescein diacetate (DCFH-DA), and phenyl-1-pyrenylphosphine (DPPP), can be used to detect cell death, intracellular ROS, and lipid peroxidation generation, respectively. These assays are often combined with OS models constructed based on zebrafish to evaluate the activity of antioxidant peptides. For example, the three assays were employed in an AAPH-exposed zebrafish to evaluate an antioxidant peptide WDVL (Trp-Asp-Val-Lys) derived from antler velvet [69]. Similarly, to evaluate the antioxidant activity of *Hippocampus abdominalis* (*H. abdominalis*) protein hydrolysate (HPH), another AAPH-exposed embryonic zebrafish was used to construct an OS model. 2′,7′-dichlorofluorescein diacetate (DCF-DA) and AO were used to detect ROS and cell death, respectively. The results showed that HPH could reduce ROS levels and cell mortality in a dose-dependent manner, indicating the protective effects of HPH [70]. In addition, it is common to detect levels of antioxidant enzymes in the zebrafish model [14]. Notably, the zebrafish model has been developed into a platform for high-throughput drug screening [18]. It is an integrated and convenient protocol with fully automated detection. High-throughput screening of antioxidant peptides based on this protocol is expected.

##### Caenorhabditis Elegans (*C. elegans*)

*C. elegans* is a non-vertebrate model organism characterized by small size, large reproduction rate, and a short life cycle [17,71]. Therefore, a population-based mean lifespan assay can be practiced in *C. elegans*. For example, functional millet bran peptides (MBPE) can overall increase the mean lifespan of *C. elegans*, especially with a 29% increase in the 12.5 µg/mL MBPE-treated group. In addition, based on the solid susceptibility of *C. elegans*, movement and reproductive rates were jointly used to synergistically evaluate the anti-aging effect of antioxidant peptides [13]. Meanwhile, the transparent nature of *C. elegans* makes fluorescent probe techniques for detecting OS still applicable. For example, the antioxidant peptide SePs was extracted from *Sepia esculenta*. DC-FHDA results showed that SePs could reduce ROS levels in the *C. elegans* OS model. Also, transgenic CF1553 nematodes, combined with green fluorescent protein (GFP), were used in this research. The expression of sod-3p::GFP in the SePs-treated group was significantly higher than that in the control group, indicating an increase in the level of expression of the antioxidant enzyme SOD-3, which was concurrently evidenced by the results of RT-qPCR [72]. The genome of *C. elegans* has been fully sequenced, and genetic manipulations on *C. elegans* can be easily implemented [73]. To sum up, multiple assays can be conducted in *C. elegans* to evaluate the activity of antioxidant peptides, which reflects an integrated evaluation system in *C. elegans*. In the future, an efficient screening of antioxidant peptides can be achieved based on the high-throughput screening platform with *C. elegans*.

##### Drosophila Melanogaster (*Drosophila*)

*Drosophila* is a classic invertebrate non-rodent model organism. Aging is closely associated with the radical reactions caused by ROS, which may be alleviated by antioxidant peptides with anti-aging potential [74]. Compared to zebrafish and *C. elegans*, *Drosophila* is employed more commonly to evaluate the anti-aging ability of antioxidant peptides. For example, crimson snapper scale peptides (CSSPs) were able to increase the average lifespan of *Drosophila*. Meanwhile, CSSPs reduced MDA and protein carbonylation (PCO) levels in *Drosophila* and increased the protein levels of total superoxide dismutase (T-SOD) and catalase (CAT), as well as the expression of related genes [75]. These results demonstrate the significant antioxidant and lifespan-extension effects of CSSPs. Apart from lifespan, healthspan is recognized as an essential indicator of anti-aging. For example, soft-shelled turtle peptides (STP) could prolong the mean lifespan of male and female *Drosophila* by 20.23% and 9.04%, respectively. STP also delayed the decline in crawling ability, enhanced intestinal barrier integrity, and provided resistance to starvation and heat stress while maintaining a certain level of food intake [76]. These results showed the extension of lifespan and healthspan effects of STP.

## 4. Molecular Mechanism of Antioxidant Peptides

### 4.1. Keap1-Nrf2/ARE Signaling Pathways

Keap1-Nrf2/ARE is a central pathway among numerous antioxidant molecular regulatory mechanisms [77] and is the most studied one for the mechanism of antioxidant peptides [78,79,80,81,82]. Keap1, as a core regulator of the pathway, can control the stability of Nrf2 based on the redox conditions in the cell to keep it exerting its antioxidant capacity reasonably. Keap1 mainly consists of a broad -complex-, tramtrack-, and bric aà brac (BTB) domain, an intervening region (IVR) domain containing multiple cysteine modification sites, and an Nrf2-binding Kelch/DGR domain that binds Nrf2 [83]. The dimerization of Keap1 is achieved with the assistance of its BTB domain by binding Cullin3 (Cul3), whereas the IVR domain bridges the BTB domain to the Kelch/DGR domain [84]. Nrf2 is a member of the Cap’n’collar (CNC) family of transcription factors, which consists of seven Neh (Nrf2 ECH homology) domains, each with a distinct function. The Neh1 CNC-bZIP domain is responsible for binding and dimerization with the small Maf (sMaf) protein; the Neh2 domain mediates the interaction with Keap1 through DLG and ETGE motifs; the Neh4, Neh5, and Neh3 domains are essential for the trans-activation of Nrf2; and the serine-rich Neh6 domain regulates the stability of Nrf2 [42]. As a specific DNA sequence, ARE can initiate the transcription of the antioxidant enzyme genes by binding to the dimerization product formed by Nrf2 and sMaf.

**Figure 1:**
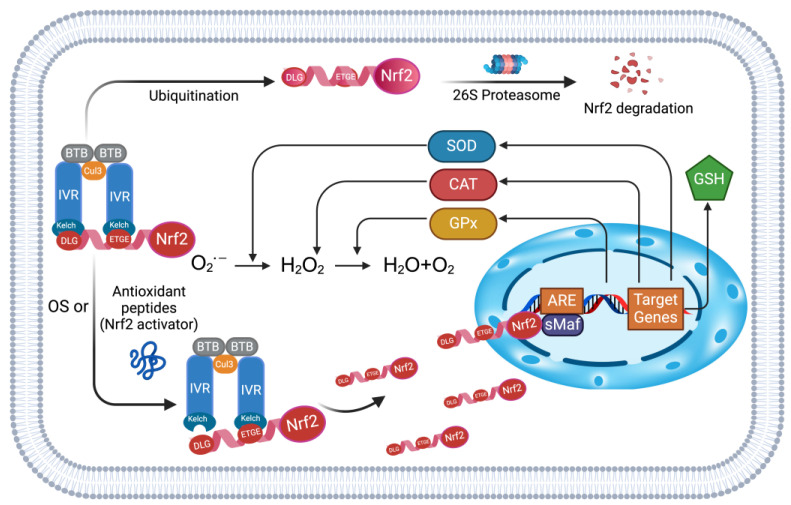
Keap1-Nrf2/ARE signaling pathway. Nrf2: nuclear factor E2-related factor 2; ARE: antioxidant response element; sMaf: small Maf; SOD: superoxide dismutase; CAT: catalase; GPx: glutathione peroxidase; BTB: broad -complex-, tramtrack- and bric aà brac; IVR: intervening region; Cul3: Cullin3; OS: oxidative stress.

There are currently two mainstream Keap1 regulation of Nrf2 stability models based on different cell redox levels: the Keap1-Cul3 dissociation model and the “Hinge and Latch” model [43]. The latter will be used below to illustrate how cells unleash their antioxidant potential through the Keap1-Nrf2/ARE pathway [85]. The scheme of the Keap1-Nrf2/ARE signaling pathway is shown in Figure 1. After dimerization, Keap1 possesses two Kelch domains that can bind to the ETGE and DLG motifs on the Nrf2 Neh domain, respectively. Among them, the ETGE motif with stronger affinity is called “Hinge”, which is used to maintain the robustness of Nrf2 binding to Keap1. The less affinity DLG, called “Latch”, regulates its state according to different cell OS levels. When cells are at normal levels, DLG maintains binding to the Kelch domain, and the assembly of Keap1, Cul3, and Rbx1 into a functional E3 ubiquitin ligase complex (Keap1-Cul3-E3) induces Nrf2 ubiquitination, which in turn is degraded via the 26s proteasome. This keeps Nrf2 at low levels under normal cellular conditions and does not induce transcription and translation of the antioxidant enzyme defense system without restriction. In contrast, under OS conditions, a large number of cysteines on the IVR domain of Keap1 are modified, leading to a conformational change of Keap1 as a whole, and the DLG motif, the “Latch”, which represents the weak binding property, is decoupled from Kelch. In this case, Keap1-Cul3-E3-induced ubiquitination degradation of Nrf2 is blocked, and the increased level of Nrf2 forms a heterodimer with sMAF, which binds to the ARE in the nucleus to initiate the transcription of a series of antioxidant enzymes (SOD, CAT, GPx, GSH) to enhance cellular antioxidant levels [84]. Overall, the Keap1-Nrf2/ARE signaling pathway maintains or releases the antioxidant potential of cells by modulating the levels of Nrf2 in response to different levels of OS in cells.

The Keap1-Nrf2 pathway is the most studied pathway among numerous molecular mechanism studies of antioxidant peptides. The primary approach is to detect the ability of antioxidant peptides to bind to the Keap1 Kelch active site (competitive binding with Nrf2) by using an in silico tool (molecular docking) [86]. The in silico results can be verified by detecting the levels of antioxidant enzymes in the downstream pathway by in vitro assays (Western blot) [79]. Three antioxidant peptides, AVPYPQ (Ala-Val-Pro-Tyr-Pro-Gln), NPIFDYVLLP (Asn-Pro-Ile-Phe-Asp-Tyr-Val-Leu-Leu-Pro), and VAPFPEV (Val-Ala-Pro-Phe-Pro-Glu-Val), were identified from brown rice. They were all restorative to the H_2_O_2_-induced OS model in Caco-2 cells, indicating their excellent antioxidant capacity. The docking results demonstrated their potent binding ability to the active site of the Kelch domain [78]. Moreover, the antioxidant peptide AFDEGPWPK (Ala-Phe-Asp-Glu-Gly-Pro-Trp-Pro-Lys) from rice bran protein showed significant oxygen radical absorbance capacity (ORAC) and DPPH^•^ scavenging ability [79]. The docking results showed the ability to occupy the active site of the Keap1 Kelch domain. The Western blot results showed that the level of Nrf2 (in the nucleus), HO-1, and SOD1 was significantly increased compared to the H_2_O_2_-treated group. These results suggest that AFDEGPWPK exerts its function by activating the Keap1-Nrf2/ARE signaling pathway.

### 4.2. Mitochondrial-Dependent Apoptosis Pathway

The term “apoptosis”, which was coined by Kerr et al. in the 1970s, refers to a programmed cellular suicide process [87]. It is believed that both external and internal pathways (stimulation by external signaling) (the mitochondrial and endoplasmic reticulum pathways) can regulate apoptosis. Overall, the basic flow of apoptosis is (a) apoptosis stimuli, (b) signaling molecular action, and (c) caspase action [88]. Mitochondria were previously known for the electron transport chain (ETC) function in ATP synthesis [89]. They are also the critical organelles in the overall process of apoptosis as the external pathway can also interplay with the mitochondria-dependent internal one to co-regulate apoptosis [90]. In recent years, research on the molecular mechanism of antioxidant peptides based on the apoptosis pathway has increased, especially after the discovery of antioxidant peptides with targeted mitochondrial functions that are more closely related to this pathway [91,92].

**Figure 2:**
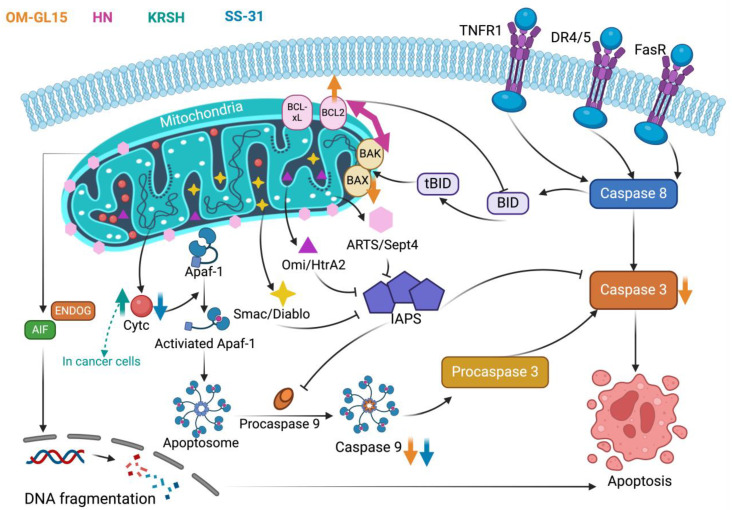
Mitochondria-dependent apoptosis pathway. TNFR1: tumor necrosis factor receptor-1; DR4/5: death receptor 4/5; FasR: Fas receptor; Caspase: cysteinyl aspartate specific proteinase; tBID: truncated BID; Apaf1: apoptotic protease-activating factor 1; AIF: apoptosis-inducing factor; ENDOG: endonuclease G; Cytc: cytochrome c; IAPs: inhibitors of apoptosis proteins.

Several essential regulators of apoptotic proteins are known as the Bcl-2 family. Members of this family include the pro-apoptotic protein Bak, located in the mitochondrial membrane, and the anti-apoptotic proteins Bcl-2 and Bcl-xL. The pro-apoptotic proteins Bax, Bid, and Bad are located in the cytoplasm. Bax can be translocated to the mitochondrial surface in response to apoptotic signals, forming a pore across the mitochondrial membrane at the mitochondrial surface. This leads to a decrease in the membrane potential, an increase in membrane permeability, and the release of apoptotic factors. The scheme of the mitochondria-dependent apoptosis pathway is shown in Figure 2. When stimulated by internal apoptotic signaling (OS), Bax level increases and binds to Bcl-2 and Bcl-xL, releasing the previously formed heterodimer Bax/Bak, with Bax and Bak forming oligomers that release Cyt c [93]. Cyt c binds to Apaf-1 in the cytoplasm, activating its nucleotide exchange activity. Activated Apaf-1 forms a whorled complex called an apoptosome, which recruits procaspase 9 to form caspase 9. Caspase 9 later activates procaspase 3 into caspase 3, thus triggering the apoptosis. Meanwhile, apoptosis signals can be mediated by apoptosis receptors, such as tumor necrosis factor receptor-1 (TNFR1), death receptor 4/5 (DR4/5), and Fas receptor (FasR). These signals activate caspase 8, which can further activate caspase 3 to trigger apoptosis. During this period, pro-apoptotic proteins, such as Smac/Diablo, Omi/HtrA2, and ARTS/Sept4, released by mitochondria can inhibit inhibitors of apoptosis proteins (IAPs) to promote apoptotic responses. In addition to the caspase-dependent apoptosis process, other pro-apoptotic factors released by mitochondria, AIF or ENDOG, can enter the nucleus and trigger DNA fragmentation, which further causes apoptosis. Concerning the external pathway, upon receiving an extracellular signaling stimulus, such as tumor necrosis factor (TNF), FAS ligand (FASL), and TNF-related apoptosis-inducing ligand (TRAIL), they bind to the respective receptors, which recruit the FAS-associated death domain (FADD) and activate caspase 8. This shears the BID of the Bcl-2 family and forms a truncated BID (tBID), which re-attracts Bax and Bak, thereby associating it with the internal mitochondrial pathway [88,93,94,95,96].

In researching the apoptotic mechanism of antioxidant peptides, detecting the level of crucial apoptotic proteins, the gene expression of the Bcl-2 family proteins, or the translocation of Cyt c in the mitochondria and cytoplasm is common. For example, a novel antioxidant peptide has been identified from the skin of *Odorrana margaretae*, OM-GL15, GLLSGHYGRASPVAC (Gly-Leu-Leu-Ser-Gly-His-Tyr-Gly-Arg-Ala-Ser-Pro-Val-Ala-Cys). OM-GL15 was proven to protect epidermal cells from ultraviolet radiation b (UVB)-induced apoptosis by up-regulating Bcl-2 and down-regulating caspase-3, caspase-9, and Bax. It suggested there is potential for OM-GL15 to be developed as a photodamage inhibitor in the cosmetic industry [92]. Similarly, Humanin (HN), a mitochondria-derived cytoprotective peptide, can inhibit apoptosis by regulating the interaction of Bcl-2 family proteins for apoptosis inhibition and possessing cellular antioxidant effects [97].

Mitochondria can generate large amounts of ROS in the cell, which is also a focused target of OS damage [98]. OS damage is an essential stimulatory signal for the induction of the internal pathway of apoptosis [95]. Thus, there might be an association between the mitochondria-dependent apoptosis pathway and OS. The importance of the apoptosis pathway in the mechanism of antioxidant peptides is highlighted by the discovery of many novel peptides that can target mitochondria to scavenge ROS. Such peptides are referred to as “targeted mitochondrial antioxidant peptides”. In most research, the mechanism of these peptides is associated with mitochondria-dependent apoptosis [91,92,97,99]. To illustrate, a synthetic KRSH peptide can target mitochondria due to its cationic Lys and Arg. It has been reported to significantly increase Cyt c release in HeLa and MCF-7 cells, suggesting that it can induce apoptosis in cancer cells and has some anticancer effects. In contrast, the results of DC-FHDA showed that the KRSH peptide could significantly scavenge intracellular and mitochondrial ROS, indicating its excellent antioxidant effects [97]. SS-31 is currently a well-known targeted mitochondria antioxidant peptide, which has been shown in vivo and in vitro to inhibit the release of Cyt c, the binding of Apaf-1 and caspase 9, and the activation of caspases. This demonstrates the potential of SS-31 to treat mitochondria in diabetic kidney disease (DKD) by inhibiting OS and apoptosis induced by HOCl-alb in podocytes [91]. In addition to DKD, SS-31 has shown good ability in treating various diseases, and the summary of cases is spread out in Section 5.2.

### 4.3. TGF-β/SMAD Signaling Pathway

**Figure 3:**
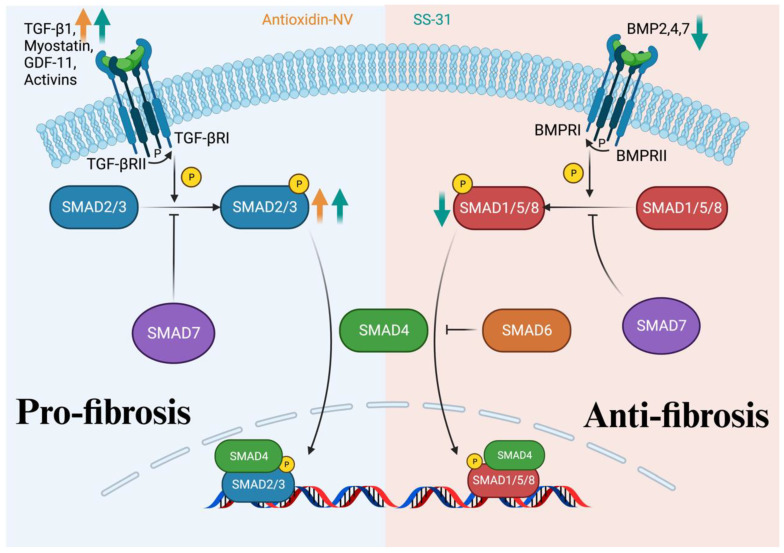
TGF-β/SMAD signaling pathway. The TGF-β and BMP, as different signaling molecules, can activate pro-fibrotic and anti-fibrotic pathways, respectively. TGF-β: transforming growth factor β; GDF-11: growth differentiation factor 11; TGF-βRI: transforming growth factor β type I receptor; TGF-βRII: transforming growth factor β type II receptor; SMAD: small mothers against decapentaplegic; BMP: bone morphogenetic protein; BMPRI: bone morphogenetic protein type I receptor; BMPRII: bone morphogenetic protein type II receptor.

The TGF-β/SMAD signaling pathway is closely associated with the formation of collagen in fibroblasts [100]. The TGF-β superfamily, including TGF-β, bone morphogenetic proteins (BMPs), activin, growth and differentiation factors (GDFs), and Nodal, can serve as the ligand for TGF-β receptors [101], and the downstream transcription factor of TGF-β is small mothers against decapentaplegic (SMAD). Based on the different transduction functions of the SMAD family proteins in the pathway, they can be classified into three groups. The first group is receptor-regulated SMAD (R-SMAD), including SMAD1/2/3/5/8, which can be activated by receptor phosphorylation; the second is the common SMAD (Co-SMAD), including SMAD4, which can bind R-SMAD to transduce the signal; and the third is inhibitory SMAD (I-SMAD), including SMAD6/7, which can inhibit the signaling pathway [102]. The scheme of the TGF-β/SMAD signaling pathway is shown in Figure 3. When TGF-β binds to the receptor, the first to be activated is TGF-β receptor II, followed by the phosphorylated activation of TGF-β receptor I. RI selectively phosphorylates activated SMAD2/3. In contrast, when BMP is the ligand, RI selectively phosphorylates activated SMAD1/5/8. The addition of SMAD4 then forms a transcriptional complex that translocates to the nucleus and initiates the transcription of target genes. SMAD7 can inhibit the transcription by suppressing the phosphorylation of SMAD2,3 or SMAD1/5/8, respectively. SMAD6 can inhibit transcriptional activity by competitively binding SMAD1/5/8 with SMAD4 [100,101,102,103,104,105].

In recent years, mechanistic research on antioxidant peptides has revolved around the TGF-β/SMAD signaling pathway. For instance, a novel antioxidant peptide, antioxidin-NV, GWANTLKNVAGGLCKMTGAA (Gly-Trp-Ala-Asn-Thr-Leu-Lys-Asn-Val-Ala-Gly-Gly-Leu-Cys-Lys-Met-Thr-Gly-Ala-Ala), was identified from the skin of *Nanorana ventripunctat*. It significantly increased TGF-β1 levels in UVB-irradiated HSF cells and hairless mice skin in a concentration-dependent manner. Also, the levels of phosphorylated SMAD2 in UVB-irradiated HSF cells were significantly increased by antioxidin-NV. In addition, antioxidin-NV was able to rescue collagen I production significantly in hairless mice and increase the abundance and density of collagen fibers after UVB irradiation in the skin of UVB-exposed mice [25]. This evidence suggests that antioxidin-NV rescues the collagens by activating the TGF-β1/SMAD2 pathway. SS-31 could also function through activating the TGF-β/SMAD pathway. Right ventricular systolic blood pressure (RVSBP) was used as a measure to evaluate the effects of SS-31 on transverse aortic constriction (TAC)-induced pulmonary arterial hypertension (PAH) in mice. The results showed that SS-31 significantly reduced the TAC-induced elevation of RVSBP. The same pattern of change was seen in the pro-fibrotic Smad3/TGF-β pathway, while the opposite was seen in the anti-fibrotic BMP-2/Smad1/5 pathway [23].

### 4.4. AMPK/SIRT1/PGC-1α Signaling Pathway

**Figure 4:**
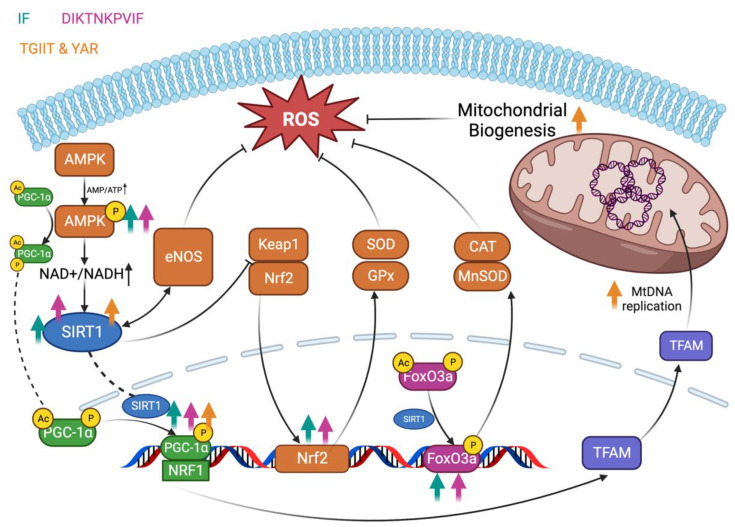
AMPK/SIRT1/PGC-1α signaling pathway. AMPK: AMP-activated protein kinase; NAD: nicotinamide adenine dinucleotide; NADH: reduced nicotinamide adenine dinucleotide; SIRT1: sirtuin 1; PGC-1α: peroxisome proliferator-activated receptor γ coactivator-1 α; NRF1: nuclear respiratory factor 1; TFAM: mitochondrial transcription factor A; Nrf2: nuclear factor erythroid 2-related factor 2; Keap1: Kelch-like ECH-associated protein 1; eNOS: endothelial nitric oxide synthase; SOD: superoxide dismutase; GPx: glutathione peroxidase; CAT: catalase; MnSOD: Manganese superoxide dismutase; FoxO3a: forkhead box class O3a; ROS: reactive oxygen species; MtDNA: mitochondrial DNA.

The AMPK/SIRT1/PGC-1α signaling pathway is closely related to mitochondrial biogenesis [106]. As the most studied molecule in the Sirtuins and the critical factor in this pathway, SIRT1 has numerous regulatory functions, including cellular senescence, cell survival and aging, and metabolism regulation [107]. In recent years, many research cases have shown that AMPK/SIRT1/PGC-1α is also responsible for the antioxidant capacity of cells. The schematic of the AMPK/SIRT1/PGC-1α signaling pathway is shown in Figure 4. When the AMP/ATP ratio in the cell increases (rapid ATP depletion through exercise), AMPK is activated by phosphorylation, which further phosphorylates and activates the PGC-1α. Meanwhile, elevated NAD^+^/NADH can activate SIRT1, which in turn deacetylates PGC-1α and activates its transcriptional activity [108]. The result is the increased expression of NRF-1, which increases the expression of TFAM and drives mtDNA replication transcription, leading to mitochondria biogenesis [109]. In addition, the deacetylation of SIRT1 can also act on FoxO3a, thereby increasing the expression of relevant target genes and achieving antioxidant effects through CAT and MnSOD [110]. Recent findings have shown that activated SIRT1 can also interplay with the Keap1-Nrf2 pathway to jointly promote antioxidant effects [111]. The interaction of SIRT1 with eNOS can also reduce the levels of ROS [110]. Overall, the activation of the AMPK/SIRT1/PGC-1α pathway can significantly increase the antioxidant capacity of cells.

In the last few years, it has been a new achievement to study the mechanism of antioxidant peptides based on the AMPK/SIRT1/PGC-1α signaling pathway [24,112,113]. For instance, an antioxidant dipeptide IF (Ile-Phe) was extracted from potato protein. It was implemented in the HFD-induced obese senescence-accelerated mouse-prone 8 (SAMP8) mouse model, and the results showed that IF could resist cardiac hypertrophy and maintain tissue homeostasis in the heart and liver sections. Interestingly, when oral IF is combined with moderate exercise (swimming), this anti-aging effect can be enhanced, also characterized by more weight loss. The upregulated pAMPK/SIRT1/PGC-1α/pFOXO3 suggested the activated AMPK/SIRT1/PGC-1α signaling pathway, which elucidated the molecular mechanism of the phenomenon as mentioned above [113]. As previously stated, sensible exercise can increase the AMP/ATP ratio to activate AMPK further. The combination of administering antioxidant peptides with scheduled training can achieve better outcomes, which provides an effective strategy for people towards weight control and anti-aging. Similarly, an antioxidant peptide, DIKTNKPVIF (Asp-Ile-Lys-Thr-Asn-Lys-Pro-Val-Ile-Phe), and IF were extracted from potato proteins. Both could significantly improve ejection fraction and fractional shortening in spontaneously hypertensive rats. The increased expression of p-AMPKα/SIRT1/PGC1α/p-Foxo3a/Nrf2/CREB in the peptide-treated group suggested that the AMPK/SIRT1/PGC-1α signaling pathway was activated, which accounted for the phenomenon in spontaneously hypertensive rats. Both peptides have therapeutic potential in spontaneous hypertension [24]. In the updated research, two novel antioxidant peptides, TGIIT (Thr-Gly-Ile-Ile-Thr) and YAR (Tyr-Ala-Arg), were identified from milk fat globule membrane (MFGM) protein. By activating the Sirt-1/PGC-1α pathway, they were shown to protect against Dex-induced OS damage in L6 cells [112]. Both peptides could inhibit mitochondria-mediated apoptosis, improve mitochondrial function, and reduce mitochondrial autophagy by directly enhancing mtDNA replication. Interestingly, the screening of antioxidant peptides with mitochondrial protective functions can be achieved by docking the candidate peptides to SIRT1. The mitochondria-dependent apoptosis and the AMPK/SIRT1/PGC-1α pathway are closely associated with the biofunction of mitochondria. The research based on these two pathways can elucidate the protective mechanism of antioxidant peptides in mitochondria (also associated with OS defense). Intended screening of antioxidant peptides based on the pathway (AMPK/SIRT1/PGC-1α) can fully unlock the mitochondrial protective potential of these peptides.

### 4.5. PI3K/Akt/mTOR Signaling Pathway

**Figure 5:**
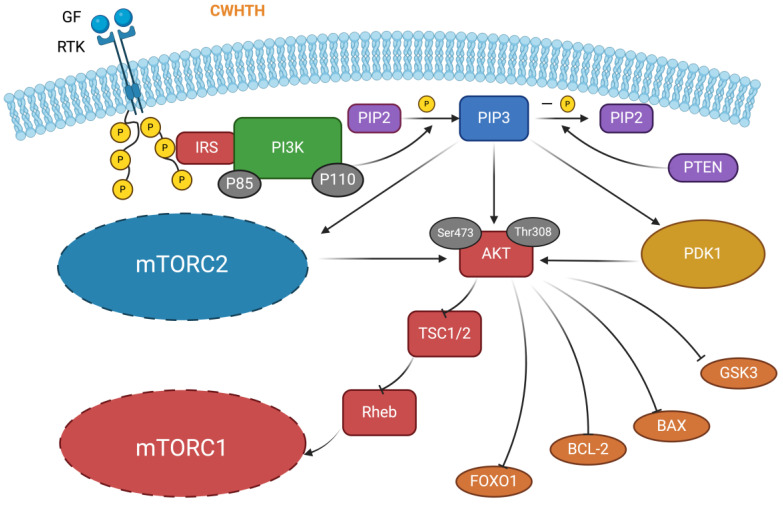
PI3K/Akt/mTOR signaling pathway. GF: growth factor; RTK: receptor protein tyrosine kinase; IRS: insulin receptor substrate; PI3K: phosphoinositide 3-kinase; PIP2: phosphatidylinositol 4,5-bisphosphate; PIP3: phosphatidyl-inositol,3,4,5 triphosphate; Akt: protein kinase B; PDK1: phosphoinositide-dependent protein kinase-1; TSC1/2: tuberous sclerosis complex 1/2; mTORC1: mammalian target of rapamycin complex 1; mTORC2: mammalian target of rapamycin complex 2; FoxO1: forkhead box protein O1; Bax: Bcl-2-associated X protein; GSK3: glycogen synthase kinase 3.

The PI3K/Akt/mTOR pathway is responsible for various cellular activities, including cell activation, growth, proliferation, and apoptosis [114]. The scheme of the PI3K/Akt/mTOR signaling pathway is illustrated in Figure 5. To elucidate the scheme of the pathway, the activation of PI3K (consisting of P85 and P110) occurs when the ligand binds to cell surface tyrosine kinase receptors (RTKs), thereby triggering its phosphorylation with the signaling adaptor protein (insulin receptor substrate, IRS). Activated PI3K can phosphorylate the phosphatidylinositol 4,5-bisphosphate (PIP2), converting it to phosphatidyl-inositol 3,4,5 triphosphate (PIP3) [115]. Meanwhile, the phosphatase and tensin homolog (PTEN), a specific PIP3 phosphatase, can dephosphorylate PIP3, reconverting it to PIP2 [116]. The activated PIP3 recruits two protein kinases, protein kinase B (Akt) and phosphoinositide-dependent protein kinase 1 (PDK1), to the plasma membrane. The Ser473 and Thr308 of Akt can be phosphorylated by the mammalian target of rapamycin complex 1 (mTORC1) and PDK1, respectively. Then, activated Akt phosphorylates tuberous sclerosis complex 2 (TSC2), inhibiting tuberous sclerosis complex 1/2 (TSC1/TSC2) complex activity. Previously, the active TSC1/TSC2 complex could inhibit the activity of Ras homolog enriched in the brain (Rheb), which in turn inhibited the activity of mTORC1. Thus, sequential inhibition enables activated Akt to activate the activity of mTORC1, further activating its downstream effectors. Meanwhile, the mammalian target of rapamycin complex 2 (mTORC2) is required for the full activation of Akt. Activated Akt inhibits forkhead box protein O1 (FOXO1), which controls cell survival. Glycogen synthase kinase 3 (GSK3) controls cell proliferation, while Bcl-2 and Bax can control apoptosis [114,117,118,119].

PI3K/Akt/mTOR activation is usually detected by analyzing the total levels of PI3K, Akt, mTOR, and their respective phosphorylated forms. For example, LWHTH (Leu-Trp-His-Thr-His) is an antioxidant peptide extracted from *Styela clava*. Based on the alanine scanning technique, a series of peptides derived from LWHTH were synthesized, among which CWHTH (Cys-Trp-His-Thr-His) possessed the most potent antioxidant activity. CWHTH showed a significant protective effect on H_2_O_2_ or APAP-treated LO_2_ cells. The enhanced levels of pPI3K/PI3K and pAkt/Akt suggested that CWHTH exerted cytoprotective effects by activating the PI3K/Akt pathway [120]. Similarly, three novel antioxidant peptides, TWLPLPR (Thr-Trp-Leu-Pro-Leu-Pro-Arg), YVLLPSPK (Tyr-Val-Leu-Leu-Pro-Ser-Pro-Lys), and KVPPLLY (Lys-Val-Pro-Pro-Leu-Leu-Tyr) were identified from *Juglans mandshurica* Maxim. By scavenging ROS, they could enhance GPx activity ATP levels and alleviate apoptosis in Aβ25-35-treated PC12 cells. The three peptides were verified to exert their cytoprotective effects through activating the PI3K/AKT/mTOR pathway [21].

### 4.6. NF-κB Signaling Pathway

**Figure 6:**
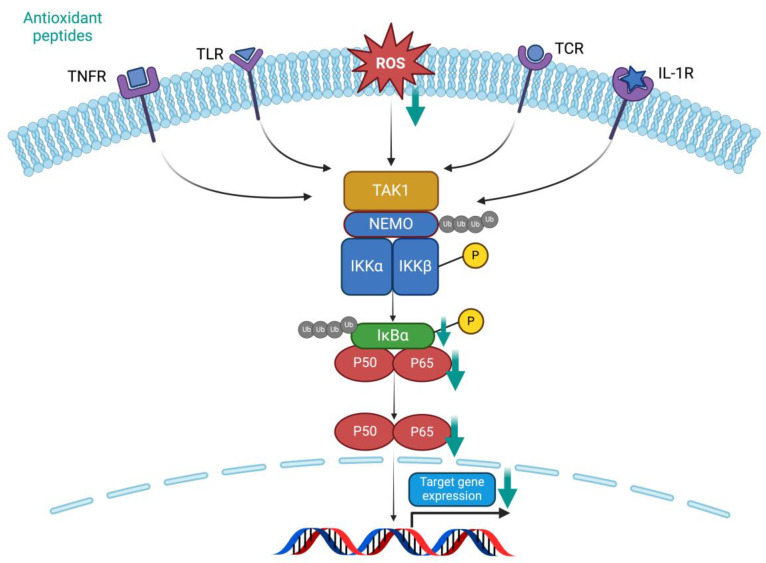
NF-κB signaling pathway. TNFR: tumor necrosis factor receptor; TLR: Toll-like receptor; TCR: T cell receptor; IL-1R: interleukin 1 receptor; TAK1: TGF-β-activated protein kinase 1; NEMO: NF-κB essential modulator; IKKα: inhibitor of κB (IκB) kinase α; IKKβ: inhibitor of κB (IκB) kinase β.

The theory underpinning the classical pathway of NF-κB suggests that the heterodimer composed of RelA (P65) and P50 of the NF-κB family is the primary inducer that triggers the transcription of downstream target genes [121]. However, under normal conditions, the P65/P50 complex is bound by the IκB protein (IκBα), which inhibits its nuclear translocation. The scheme of the NF-κB signaling pathway is demonstrated in Figure 6. The formation of TGF-β-activated protein kinase 1/inhibitor of κB (IκB) kinase (TAK1/IKK) complex is induced by all kinds of binding between ligands and their corresponding receptors (TNFR1, TLR, TCR, IL-1R1). OS can also be the stimuli signaling. IKK consists of two homologous catalytic subunits, IKKα and IKKβ, and a regulatory subunit, NF-κB essential modulator (NEMO) (also known as IKKγ). IKK further leads to the phosphorylation of IκBα and subsequent ubiquitination degradation. As a result, the free P65/P50 complex is translocated to the nucleus, inducing the transcription of pro-inflammatory or apoptosis signaling molecules, further leading to cellular injury [121,122,123].

Since the NF-κB pathway is closely linked to pro-inflammatory, apoptosis, or fibrotic pathways, it is often combined with other essential biomarkers to illustrate the molecular mechanism underlying antioxidant peptides jointly. The abovementioned antioxidin-NV was shown to reduce the levels of IkBα and p65 in UVB-irradiated HaCaT cells in a concentration-dependent manner, suggesting its inhibitory effect on the NF-κB pathway. Meanwhile, it accounted for the previous concerns about the decrease in levels of IL-6 (an inflammatory biomarker) mediated by antioxidin-NV, which is involved in TLR. Antioxidin-NV can be developed as an anti-photoaging agent [25]. Similarly, a rapeseed protein-derived antioxidant peptide (RAP), YWDHNNPQIR (Tyr-Trp-Asp-His-Asn-Asn-Pro-Gln-Ile-Arg), attenuated the levels of phosphorylated P65 in diabetic mice, suggesting an inhibition of the NF-κB pathway, which also serves as one of the mechanisms by which previous RAPs alleviated diabetic renal fibrosis [124]. In addition, an antioxidant peptide, VKAGFAWTANQQLS (Tyr-Trp-Asp-His-Asn-Asn-Pro-Gln-Ile-Arg), derived from tuna backbone, enhanced its cell-penetrating ability by binding to the cell-penetrating peptide GRKKRRQRRRPPQ (Gly-Arg-Lys-Lys-Arg-Arg-Gln-Arg-Arg-Arg-Pro-Pro-Gln). A similar study alleviated the inflammatory response in necrotizing enterocolitis by scavenging ROS and inhibiting the NF-κB pathway [125].

## 5. Application of Antioxidant Peptides

### 5.1. Antioxidant Peptides in Food Manufacture

Antioxidant peptides can be the main ingredient of functional foods to ensure the nutritional value of products. As small molecules, antioxidant peptides have good absorption, utilization, and solubility in acidic media, and they are often developed as functional beverages in healthcare products [126,127]. Additionally, OS leads to the excessive production of ROS in food products or living tissues (meat products), which can further cause the oxidation of lipids and proteins. In this case, food spoilage can occur with the color or odor diminishing. Antioxidant peptides can function as a food additive to stabilize and improve the quality of food products, thus improving sensory acceptance (odor and color). Table 1 lists the applications of antioxidant peptides in food manufacture.

Table 2 gives specific products in the food industry with the roles of the peptides analyzed. It can be seen that antioxidant peptides can be the main ingredient in functional products (beverages or soups). In addition, the flavor enhancement provides a basis for the commercial application of antioxidant peptides. To sum up, these examples illustrate the wide range in applications of antioxidant peptides throughout food manufacture, providing the basis for the development of the food industry.

### 5.2. Antioxidant Peptides in Therapy

OS occurs when the ROS exceeds the antioxidant defense system in the body, causing the accumulation of peroxides, which in turn leads to various diseases, such as neurodegenerative diseases [134], cardiovascular diseases [135], and renal diseases [136]. Bioactive peptides have multifunctionality, high membrane permeability, and low toxicity. The US Food and Drug Administration (FDA) has recently approved several peptides as therapeutic agents [137,138,139]. Nowadays, antioxidant peptides have provided a new therapy for various diseases.

Neurodegenerative diseases are caused by the misfolding of proteins/peptides, resulting in the intertwining of extracellular amyloid-β plaques and neural progenitor fibers inside neurons, which consequently disrupts the signaling between neurons and finally leads to neuronal death [140]. Alzheimer’s disease (AD) and Parkinson’s disease (PD) are the typical disorders of neurodegenerative diseases. Intracerebral OS is one of the causes and secondary manifestations of AD. Various OS markers in the brain’s frontal, parietal, hippocampal, and cortical parts are elevated compared to normal subjects [141]. Therefore, the antioxidant activity of the food-derived peptides may treat AD. Moreover, AD is partly caused by the loss of ACh catalyzed by AChE and BuChE [142]. So, antioxidant peptides with inhibition of AChE and BuChE cholinesterase activity or with structures and active centers similar to Ach have more potential for treating AD [143]. For example, antioxidant peptide fractions from pea protein can scavenge free radicals and inhibit lipid peroxidation. These fractions also contain around 30% activity of both AChE and BuChE, which increases their potential as candidates for treating AD [144]. Furthermore, neuroinflammation in the brain releases various pro-inflammatory cytokines such as tumor necrosis factor-α (TNF-α), interleukin-6 (IL-6), and interferon-γ (IFN-γ). They can induce neurotoxicity, which is one of the pathological causes of AD [145]. An antioxidant peptide from *Salvia hispanica* showed a high scavenging rate of ROS, TNF-α, and IL-6 in HMC3 microglia. This result indicated that the peptide possessed significant antioxidant and anti-inflammatory activity against this neuronal cell, which is an essential basis for being a therapeutic candidate for AD [146]. In conclusion, for complex diseases with diverse pathological causes, such as AD, the antioxidant peptide is an essential therapeutic agent for targeting multiple etiologies of AD.

OS is a significant manifestation of cardiovascular diseases [147]. In vascular blood, OS markers (H_2_O_2_, MDA) significantly increase in multifactorial causative diseases such as hypertension. Antioxidant peptides can alleviate these diseases as secondary therapeutic agents. Two antioxidant peptides, YAVT (Tyr-Ala-Val-Thr) and YLL (Tyr-Leu-Leu), identified from the alga *Eucheuma cottonii*, exhibited significant activity of scavenging DPPH^•^, HO^•^, and O_2_^•−^. Meanwhile, the activity of SOD, GPx was enhanced. Also, both peptides effectively inhibited H_2_O_2_-induced apoptosis in HUVECs [148]. These suggest their potential in treating cardiovascular diseases. As mentioned before, OS markers in the plasma of hypertensive patients were increased, which was associated with the production of superoxides catalyzed by NAD(P)H oxidase, while the efficiency of nitric oxide (NO) to promote vascular smooth muscle diastole was crowded out, thus promoting an increase in blood pressure. Therefore, targeting NAD(P)H oxidase and selectively inhibiting its activity would enable an efficient treatment of cardiovascular diseases like hypertension. Currently, targeting antioxidant peptides CRPPR (Cys-Arg-Pro-Pro-Arg) and CSGMARTKC (Cys-Ser-Gly-Met-Ala-Arg-Thr-Lys-Cys) have been discovered. They can bind to another peptide, gp91ds, in the aortic and cardiac vascular systems to inhibit NAD(P)H oxidase activity and reduce the elevation of OS markers. Meanwhile, the bioavailability of NO is substantially increased [149]. Targeted antioxidant peptides have robust specificity and can be effective in treating diseases with fewer side effects. Currently, a representative targeted antioxidant peptide is SS-31.

The Szeto-Schiller (SS) peptide family is a class of amphipathic tetrapeptides rich in the anionic phospholipid cardiolipin (CL) with the ability to target mitochondrial membranes, with therapeutic effects against a wide range of mitochondrial diseases. SS-31(H-D-Arg-DMT-Lys-PHE-NH_2_), is known as elamipretide. As an essential member of the SS peptide family, the SS-31 peptide possesses free radical scavenging ability due to dimethyl tyrosine. It can be directly enriched in the inner mitochondrial membrane independent of its potential alteration, whereas most mitochondrial targeting molecules depend on potential alteration. Therefore, SS-31 can efficiently target mitochondria to scavenge ROS and treat relevant diseases. SS-31 has a wide range of applications in the treatment of various diseases, especially in the protection of cardiolipin from OS attack, which effectively protects cardiac function and simplifies the treatment of diseases with complex pathologies. Among the cardiolipin-protecting agents, it is the “first-in-class” one [150]. Table 2 summarizes the application of antioxidant peptides in treating diseases, especially SS-31, which is widely used.

**Table 2:** Applications of antioxidant peptides in therapy.

Antioxidant Peptides	Target Diseases	Mechanisms	References
SS-31	Peripheral artery disease (PAD)	Inhibited the AKT-mTOR pathway to restore impaired autophagic flux; reduced levels of p-AKT p-mTOR; reduced ROS levels	[151]
	Renal fibrosis and chronic renal failure (CRF)	Protected mitochondrial membrane potential, ATP production, mtDNA copy number, MnSOD activity; reduced ROS levels	[152]
	Alzheimer’s disease (AD)	Lowered ROS and Aβ levels, protecting mitochondrial homeostasis and synaptic integrity	[153]
	Type 2 diabetes (T2D)	Inhibited lipid peroxidation and ATP production; protected mRNA expression of dynamic mitochondrial genes; reduced mitochondrial dysfunction	[154]
	Myocardial ischemia reperfusion injury (MI/RI)	Inhibited mPTP opening and ROS production; alleviated apoptosis in cardiomyocytes; reduced inflammatory cells; maintained integrity of mitochondrial function	[155]
	Pulmonary arterial hypertension (PAH)	Increased levels of antioxidant markers HO-1, NQO-1, GR, GPx; decreased levels of OS markers of NOX-1, NOX-2, and oxidized proteins; decreased levels of inflammatory markers of MMP-9, TNF-α, and iNOS	[23]
A peptide from Ziziphus jujuba	Alzheimer’s disease	Inhibited activity of AChE and BuChE	[156]
A peptide from yellow field pea proteins	Alzheimer’s disease	Inhibited activity of AChE and BuChE; inhibited lipid peroxidation; scavenged free radicals	[144]
Cocaine and Amphetamine Regulated Transcript (CART) peptide	Parkinson’s disease	Protected cellular mtDNAs; inhibited lipid peroxidation; reduced ROS levels	[157]
Peptides from Salvia hispanica	Alzheimer’s disease/Parkinson’s disease	Reduced levels of ROS, TNF-α, and IL-6 in HMC3 cells	[146]
Two peptides from Eucheuma cottonii	Cardiovascular diseases	Scavenged free radicals of DPPH^•^, HO^•^, O_2_^•−^; Increased activity of SOD and GPx; inhibited H_2_O_2_-induced apoptosis in HUVECs	[148]
Vascular-targeting peptides CRPPR and CSGMARTKC	Hypertension	Linked to the antioxidant peptide gp91ds; inhibited activity of NAD(P)H oxidase; reduced production of superoxide;	[149]
A hybrid peptide LL-37-TP5	Intestinal inflammation	Neutralized LPS; inhibited OS levels; inhibited NF-κB signaling pathway; decreased levels of TNF-α, IFN-γ, and IL-6; increased expression of ZO-1 and occludin; reduced permeability in the jejunum	[158]
Peptides from wheat germ	Celiac disease (CD)	Reduced ROS levels; increased activity of CAT, GR, GPx, and GSH)/GSSG levels; activated keap1/Nrf2 signaling pathway	[159]
DR7dA [DHNNPQ (D-Ala) R-NH_2_]	Pulmonary fibrosis (PF)	Attenuated TGF-β1-induced fibrogenesis and ameliorated bleomycin-induced fibrosis; inhibited extracellular matrix deposition and MAPK signaling pathway	[160]

Notes: “Mechanism” includes changes in relevant indicators or signaling pathways addressed in the original research. p-AKT: phosphorylated AKT; p-mTOR phosphorylated mTOR; MnSOD: manganese superoxide dismutase; Aβ: β-amyloid; mPTP: mitochondrial permeability transition pore; ROS: reactive oxygen species; ATP: adenosine triphosphate; HO-1: heme oxygenase-1; NQO-1: NADPH quinone oxidoreductase-1; GR: glutathione reductase; GPx: glutathione peroxidase; OS: oxidative stress; NOX-1: NADPH oxidase-1; NOX-2: NADPH oxidase-1; MMP-9: matrix metalloproteinase 9; TNF-α: tumour necrosis factor-alpha; iNOS: inducible nitric oxide synthase; ZO-1: zonula occludens-1; IL-6: interleukin 6; IFN-γ: interferon-gamma; LPS: lipopolysaccharide; HMC3 cells: human microglial clone 3 cells; NF-κB: nuclear factor kappa B; CAT: catalase; GSH: glutathione; GSSG: oxidized glutathione; TGF-β1: tumor growth factor-β1.

It can be seen that both food-derived and synthetic antioxidant peptides have the potential for therapy when combined with the cases mentioned above and the examples given in Table 2. Prevention is the key to those serious diseases secondary to OS, such as AD, for which there is currently no fully curative program. Food-derived antioxidant peptides may be less expensive and safer than synthetic peptides for daily use. It is a proven way to supplement food-derived antioxidant peptides as a preventive approach. In contrast, there is a trend toward developing synthetic antioxidant peptides as targeted drugs, with their applications focusing on the pharmaceutical field. The SS-31 peptide is a prominent product among the targeted mitochondrial drugs to scavenge ROS. However, more research is still required to prove the safety of SS-31 in humans before unlocking its potential in clinical therapy [161].

### 5.3. Antioxidant Peptides in the Cosmetic Industry

The aging phenomenon in human skin results in skin that appears dull and wrinkled partly because of the excess of free radicals in the human body, which leads to normal cell dysfunction. Antioxidant peptides can function by scavenging free radicals with fast absorption and good stability. Therefore, skin aging can be delayed, which is the basis for its application in the cosmetic industry.

Two crucial antioxidant peptides, L-carnitine and GSH, were applied to the cosmetic industry. L-carnitine is a dipeptide composed of α-alanine and L-histidine, which can exert its potent antioxidant activity by scavenging free radicals, chelating metal ions, and neuromodulation to exert anti-wrinkle effects [162]. In addition, L-carnitine has been reported to significantly increase the replicative lifespan of fibroblasts, thus exerting its anti-aging function [163]. GSH is a tripeptide composed of γ-glutamyl-L-cysteine glycine. GSH is a commonly used antioxidant positive control due to its powerful antioxidant effects [164], the basis for making skin care products. By mixing with other ingredients, such as mud, the antioxidant activity of GSH can be enhanced, reducing wrinkles, increasing elasticity, and reducing the pigmentation of the skin [165]. In addition, GSH can convert true melanin into melanophilic pigments and inhibit melanin formation by binding copper to inhibit tyrosine activity. For this reason, GSH is commonly used in the cosmetic field as a skin-brightening agent to provide a whitening effect. Applying GSH in the cosmetic field goes far beyond being an active ingredient. As the first line of defense of endogenous antioxidants in the human body, GSH can be regarded as an essential marker indicating OS status and serves as a quantitative toxicological assessment. To illustrate, GSH has been applied in a skin-liver model to quantify the allergic toxicity of topical cosmetics to the liver [166]. Nowadays, several fermentation techniques have solved the common problem of low GSH production yield [167,168]. It can meet the requirements of large-scale industrial production and has a wide range of industrial applications in the cosmetic field.

Collagen peptides are the widely used antioxidant peptides in the cosmetic industry. Collagen peptides can enhance the ability to absorb, hold, and retain skin moisture [169]. More importantly, collagen peptides have good anti-aging activity on the skin, which is the most crucial concern of the cosmetic industry nowadays. The collagen peptides extracted from the scales of milkfish, one of the most extensive aquaculture products worldwide, have been tested to exhibit good hygroscopic and moisturizing properties, skin anti-aging capacity, and the ability to inhibit tyrosinase activity and melanin. In addition, its toxicity was within the controlled range by cell cycle analysis by flow cytometry [170]. It proved the multiple protective effects of collagen peptides on the skin, with its safety guaranteed. The formation of wrinkles is a remarkable feature of skin aging. The loss rate of collagen in human skin accelerates with aging, thus causing a decrease in the synthesis of elastin fibers. Collagen peptides can bind to receptors on fibroblast membranes, promoting the formation of new elastin fibers for anti-wrinkle and anti-aging effects. The mechanism of anti-aging underlying collagen peptides has been a critical research direction. In another study, chicken-derived collagen peptides were shown to reduce skin OS levels, inhibit the expression of transcription factor AP-1, activate the TGF-β/SMAD signaling pathway, which promotes collagen synthesis, and inhibit the expression of MMP-1/3, which inhibits collagen degradation [171]. Thus, collagen peptides can exert their effect.

Collagen peptides are generally produced by the enzymatic digestion of collagen. Therefore, the source of collagen and the enzymatic digestion process are critical factors for the industrial production of collagen peptides. Traditional sources of collagen peptides have predominantly been derived from pigs and cattle. However, collagen peptides extracted from aquatic animals have the potential for broader applications in the future. Marine collagen peptides exhibit higher safety, intestinal bioavailability, and homology with human collagen [172]. More importantly, the byproducts of the production process, such as aquatic fish scales and offal, serve as a crucial source of collagen peptides. This makes them a more ecofriendly option for industrial production. Table 3 shows the application of collagen polypeptides of aquatic fish origin in production, which shows the versatility of their functions and deserves to be explored more deeply in cosmetics. The specific enzymatic conditions are also given for research reference.

Collagen peptides are currently prevalent in the cosmetic industry. Discovering novel collagen sources means discovering possible new activity. Furthermore, some by-products of animal processing, or waste, may hold great potential for collagen peptide discovery in a more environmentally friendly way. In addition, the optimization of various collagen enzymatic processes is also a worthy direction for future research. New studies have found that some amphibian-derived antioxidant peptides have sound effects on preventing photodamage [178], and antioxidant peptides of precious plant origin have tanning and whitening effects [179]. Some novel synthetic antioxidant peptides can exert anti-aging effects on the skin by increasing the expression of type I collagen and decreasing the expression of matrix metalloproteinase-1 (MMP1) [180]. These examples show that besides L-creatine, GSH, and collagen peptides, various other sources of antioxidant peptides have great potential for application in the cosmetic industry and deserve to be explored.

**Figure 7:**
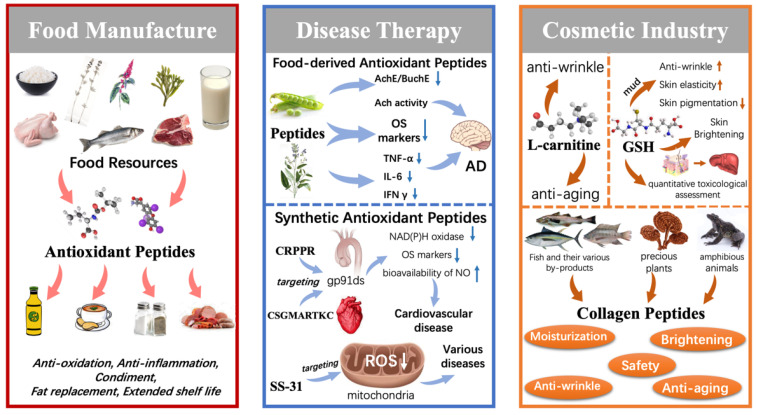
Application of antioxidant peptides. AchE: acetylcholinesterase; BuchE: butyrylcholinesterase; OS: oxidative stress; TNF-α: tumour necrosis factor-alpha; IL-6: interleukin-6; IFN γ: interferon-gamma; AD: Alzheimer’s disease; CRPPR: Cys-Arg-Pro-Pro-Arg; CSGMARTKC: Cys-Ser-Gly-Met-Ala-Arg-Thr-Lys-Cys; SS-31: elamipretide; ROS: reactive oxygen species; GSH: glutathione.

The application of antioxidant peptides is summarized from three perspectives: food manufacture, therapy, and the cosmetic industry. Figure 7 gives an overview of the specifics. In food manufacture, what is taken from the food can be used for the food. Antioxidant peptides can be obtained from various food resources and processed into food products with diverse functions. In therapy, both food-derived and synthetic antioxidant peptides have the potential to treat various diseases. In the cosmetic industry, L-carnitine, GSH, and collagen peptides share the activity preferred by the cosmetic industry.

## 6. Conclusions and Outlooks

In conclusion, this review has summarized the novel technologies in the efficient screening of antioxidant peptides from a new vision. The evaluation models, including in vitro cellular models and in vivo rodent and non-rodent models, are reviewed systematically. Moreover, six signaling pathways, Keap1-Nrf2/ARE, mitochondria-dependent apoptosis, TGF-β/SMAD, AMPK/SIRT1/PGC-1α, PI3K/Akt/mTOR, and NF-κB, are introduced to clarify the distinct mechanism of action underlying antioxidant peptides. Finally, the multidimensional applications of antioxidant peptides in food production, therapy, and the cosmetics industry are summarized.

The efficient screening of peptides with prominent activity should be the focus to expand the widespread use of antioxidant peptides. For this purpose, a high-throughput screening platform can be developed based on non-rodent models. The trend is now toward the use of novel technologies for the efficient screening of antioxidant peptides.

Molecular mechanisms elucidate the nature of the activity underlying antioxidant peptides. On the other hand, the molecular mechanisms can be used in reverse to screen the peptides with prominent activity. It is straightforward, as the PPI of Keap1-Nrf2 can be used to screen direct inhibitory peptides. Another case is the screening of bioactive peptides by docking SIRT1, as shown in Section 4.4.

Finally, this paper compares synthetic antioxidant peptides and natural food-derived antioxidant peptides from two aspects, mainly including the screening of the PPI-based inhibition of Keap1-Nrf2 and its application in disease treatment. It is interesting to compare the two and learn from each other to promote more in-depth research in bioactive peptides.

## Figures and Tables

**Table 1:** The application of antioxidant peptides in food manufacture.

Products	Peptide Source	Function	Classification	References
A non-dairy germinated amaranth-based functional beverage	*Amaranthus hypochondriacus* L.	Anti-oxidation; anti-hypoglycemia	Beverage	[126]
A beverage consists of antioxidant peptides and *Acanthopanax senticosus* extract	Bovine milk casein	Anti-oxidation; anti-depression	Beverage	[127]
An egg white-based berry-flavored and collagen peptide-enriched dairy replacement	Bovine collagen	Anti-oxidation; better sensory acceptance (color, flavor, and taste formation)	Beverage	[128]
A prebiotic soursop whey beverage processed by high-intensity ultrasound	Bovine milk casein	Anti-oxidation; antidiabetic; anti-hypertensive; anticancer	Beverage	[129]
A beverage containing *Lupinus angustifolius* protein hydrolysates	*Lupinus angustifolius* protein	Anti-oxidation; anti-inflammation; lowering cholesterol levels and inhibiting atherosclerosis	Beverage	[130]
*Fucus vesiculosus* soup	Aqueous extract of *Fucus vesiculosus*	Anti-oxidation; hypercholesterolemia lowering effect	Soup	[131]
Chicken essence enriched with carnosine	Chicken meat	Anti-oxidation; promoting recovery of hematopoietic inhibition	Condiment	[132]
Fish collagen hydrolysate as a fat replacer in buffalo patties	Fish collagen	Anti-oxidation; low fat	Meat products	[133]
Beef products with added rice protein hydrolysates	Rice protein	Anti-oxidation with extended shelf-life	Meat products	[133]

**Table 3:** Application of antioxidant collagen peptides derived from fish.

Peptide Source	Enzymatic Conditions	Function	References
*Prionace glauca*	Alcalase, 55 °C, pH 8.0, enzyme/protein ratio of 1:20 *w*/*w*, 3 h	Increasing the expression of collagen type I mRNA	[173]
*Oreochromis niloticus*	A complex enzyme of neutral protease and papain, 50 °C, 5 h	Promoting wound healing	[174]
*Oncorhynchus keta*	Complex enzyme, 40 °C, pH 8.0, 3 h	Promoting angiogenesis and wound healing	[175]
*Thunnus albacares*	Alcalase (2%, *v*/*v*), pH 8.0, 55 °C, 3 h	Optimizing antioxidant activity	[176]
*Gadus chalcogrammus*	Trypsin, pH 8.0, 50 °C, enzyme/protein ratio of 0.6% (*w*/*w*), 4 h	Chelating dietary minerals	[177]

## Data Availability

No new data were created in this study. Data availability sharing is not applicable.
